# Longitudinal Stent Deformation at the Proximal Segment of the Left Main Coronary Artery Caused by a Stuck OCT Catheter: Case Report and Review of Literature

**DOI:** 10.31083/j.rcm2504119

**Published:** 2024-03-28

**Authors:** Ming Zhang, Xietian Pan, Yabin Wang, Shuo Yin, Peng Bai, Lei Gao

**Affiliations:** ^1^Senior Department of Cardiology, the First Medical Center of PLA General Hospital, 100853 Beijing, China; ^2^Senior Department of Cardiology, the Sixth Medical Center of PLA General Hospital, 100853 Beijing, China; ^3^Senior Department of Cardiology, the Second Medical Center of PLA General Hospital, 100853 Beijing, China; ^4^Department of Cardiology, Gu'an County Hospital of Traditional Chinese Medicine, 065500 Langfang, Hebei, China; ^5^Senior Department of Cardiology, Yulin First Hospital, 719000 Yulin, Shaanxi, China

**Keywords:** longitudinal stent deformation, optical coherence tomography, complication

## Abstract

It has been rarely reported that a stuck optical coherence tomography (OCT) catheter can lead to longitudinal stent deformation (LSD). This complication can result in incomplete stent apposition and dissection after stent implantation. In this study, we present a case where a bailout stent was implanted in the distal segment of the left anterior descending artery (LAD) after longitudinal stent deformation caused by a stuck OCT catheter. This approach was taken to prevent acute stent thrombosis, subacute stent thrombosis, in-stent restenosis (ISR), and death. The patients were followed up for one year, and no adverse events were observed.

## 1. Introduction

Coronary longitudinal stent deformation (LSD) is a rare complication occurring in approximately 0.2%–0.68% of cases [1, 2]. LSD refers to the local distortion of an implanted coronary stent along its longitudinal axis during coronary intervention, leading to a shortening of the stent by more than 2 mm [3]. Angiography reveals a ring-shaped segment at the site of LSD, caused by overlapping stent beams, often referred to as the “organ effect” or “accordion effect”. Several independent risk factors have been identified for LSD, including complex coronary arteries (with features such as coronary artery calcification, coronary artery tortuosity, long lesions, and open lesions), prolus element stents, postdilatation balloons, and intravascular imaging techniques like intravenous ultrasound (IVUS) [4]. IVUS or optical coherence tomography (OCT) can accurately detect stent beam collapse, malapposition, dissection, and thrombus formation at the LSD segment. It is important to note that not all cases of LSD are benign, as approximately 14% of patients experience serious adverse events including the need for emergency surgery due to a stuck device, acute stent thrombosis, subacute stent thrombosis, in-stent restenosis (ISR), and even death [5]. Limited clinical studies suggest that re-implantation of a stent or postdilatation treatment following the occurrence of LSD may reduce the incidence of adverse events.

## 2. Materials and Methods

The keywords “longitudinal stent deformation”, “longitudinal stent compression”, “stent deformation”, “concertina”, “stent shortening”, and “accordion” were used for literature search on PubMed. Our study was conducted based on previous studies and a review of LSD cases (Table 1, Ref. [1, 6, 7, 8, 9, 10, 11, 12, 13, 14, 15, 16, 17, 18, 19, 20, 21]). Furthermore, we thoroughly examined the existing literature on LSD.

**Table 1.** **Longitudinal stent deformation case reports**.

Author	Patient’s Age	Causes	LSD stents	LSD position	Disposal methods	Follow-up
Hernández-Matamoro *et al*. [16]	76	Balloon of stent	Resolute Onyx	Proximal	Stent	Uk
Kobayashi *et al*. [9]	73	Uk	Platinum chromium	Proximal	No	18 months: VST
Si *et al*. [6]	60	Balloon	Promus Element	Proximal	Post-dilation	12 months: normal
Buysschaert *et al*. [15]	74	Balloon	Promus Element	Proximal	No	24 months: VST
Aung *et al*. [14]	41	Balloon	Promus Element	Proximal	Post-dilation	Uk
Foerst *et al*. [13]	69	Balloon	Promus Element	Proximal	Post-dilation	4 days: normal
Leong *et al*. [11]	69	Rotawire	Synergy	Distal	Stent	Uk
Panoulas *et al*. [10]	78	Buddy wire	Resolute Onyx	Proximal	Stent	Uk
	85	Wire	Resolute Onyx	Proximal	Stent	Uk
Demir *et al*. [18]	67	Pressure wire	Xience Pro	Proximal	Stent	Uk
	54	Pressure wire	Resolute Onyx	Proximal	Stent	Uk
Vijayvergiya *et al*. [17]	70	Balloon	Promus Element	Proximal	Post-dilation	3 ddays: subacute ST
Cuenza *et al*. [7]	57	Uk	Resolute Onyx	Proximal	Stent	Uk
Yıldırım *et al*. [8]	60	EBU 3.5	Promus Element	Proximal	CABG	5 days: normal
Yew *et al*. [12]	60	Balloon	Promus Elemen	Proximal	Stent	3 months: normal
Shannon *et al*. [20]	55	IVUS	Promus Element	Proximal	Post-dilation	Uk
	62	Balloon	Promus Element	Proximal	No	Uk
	68	Wire	Promus Element	Proximal	Stent	Uk
	70	Wire	Promus Element	Proximal	Stent	6 months: normal
	76	Balloon	Promus Element	Proximal	Stent	Uk
Hanratty *et al*. [19]	50	AL	Promus Element	Proximal	Stent	Uk
	72	IVUS	Biomatrix	Proximal	Stent	Uk
	78	EBU	Resolute Integrity	Proximal	Post-dilation	Uk
Cosgrove *et al*. [21]	67	Reverse RG3	Synergy	Distal	Stent	12 months: normal
Williams *et al*. [1]	50	Balloon	Promus Element	Proximal	Stent	4 months: normal
	63	Balloon	Promus Element	Proximal	Post-dilation	Uk
	55	Catheter	Promus Element	Proximal	Post-dilation	4 months: normal
	63	Balloon	Promus Element	Proximal	Stent	Uk
	83	Catheter	Biomatrix	Proximal	No	2 months: subacute ST
	72	Catheter	TAXUS	Proximal	Stent	6 months: normal
	55	Balloon	Promus Element	Proximal	Stent	Uk
	63	Catheter	Promus Element	Proximal	Stent	Uk
	82	Balloon	Endeavor	Proximal	Stent	Uk

uk,unknown; CABG, coronary artery bypass grafting; ST, stent thrombosis; VST, very late stent thrombosis; AL, Amplaze guiding; LSD, longitudinal stent deformation; IVUS, intravenous ultrasound.

## 3. Case Report

A 42-year-old male patient was admitted to the hospital and diagnosed with coronary artery disease, acute inferior wall myocardial infarction, and type 2 diabetes mellitus. Prior to admission, he underwent coronary angiography at a local hospital and thrombus aspiration was performed in the blocked circumflex artery without stent implantation. On admission, the examination revealed sinus rhythm with the presence of Q waves in leads II, III, and Augmented Vector Foot (aVF). Cardiac ultrasound demonstrated mild tricuspid valve regurgitation and a left ventricular ejection fraction (LVEF) of 63%.

**Fig. 1.**
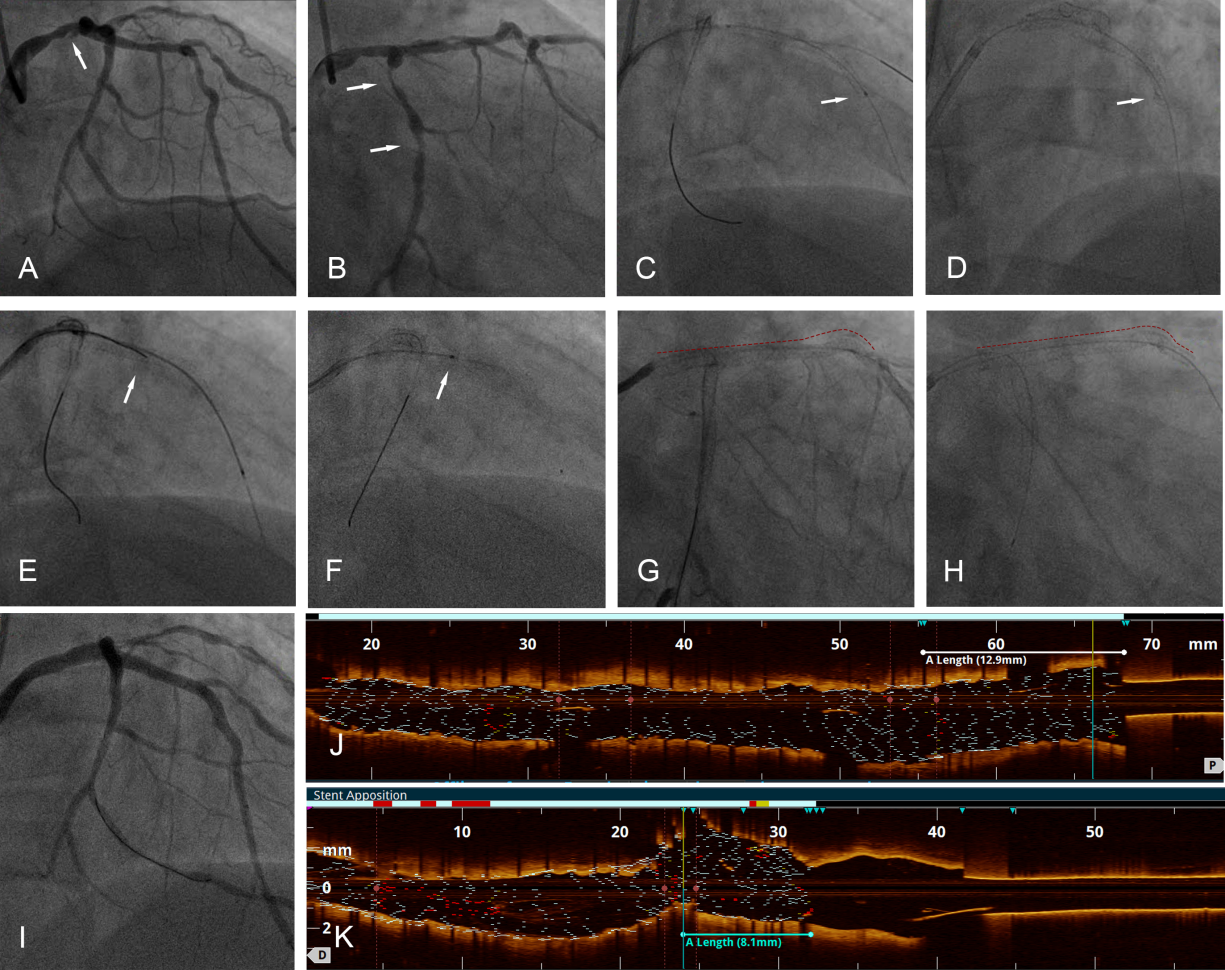
**Percutaneous coronary intervention procedure**. (A) CAG showed borderline lesions and suspicious niche in the distal segment of the LM (white arrow). (B) CAG showed diffused stenosis in the proximal to the middle segment of the LCX (white arrow). (C) Image of the stents after implantation of one LCX stent and two LAD stents, the distal segment of the LAD stents seemed properly expanded in CAG (white arrow). (D) CAG showed the lumen at the edge of the stent and at the middle segment of the LAD decreased in size (white arrow). (E) Wireless OCT catheter in LAD (white arrow indicates retracted guidewire). (F) OCT catheter stuck in retracting (white arrow indicates the position of stuck). (G) LM-LAD stents before stuck of OCT catheter, red dashed line depicted the length of LM-LAD stents. (H) Shortened LM-LAD stents after retraction of stuck OCT catheter, red dashed line depicted the length of LM-LAD stents. (I) CAG after bailout stent implantation in LAD. (J) The length from the ostium of the LAD to the stent of the LM before LSD is 12.9 mm. (K) The length from the ostium of the LAD to the stent of the LM after LSD is 8.1 mm. CAG, coronary angiography; LM, left main artery; LCX, circumflex artery; LAD, left anterior descending artery; OCT, optical coherence tomography.

**Fig. 2.**
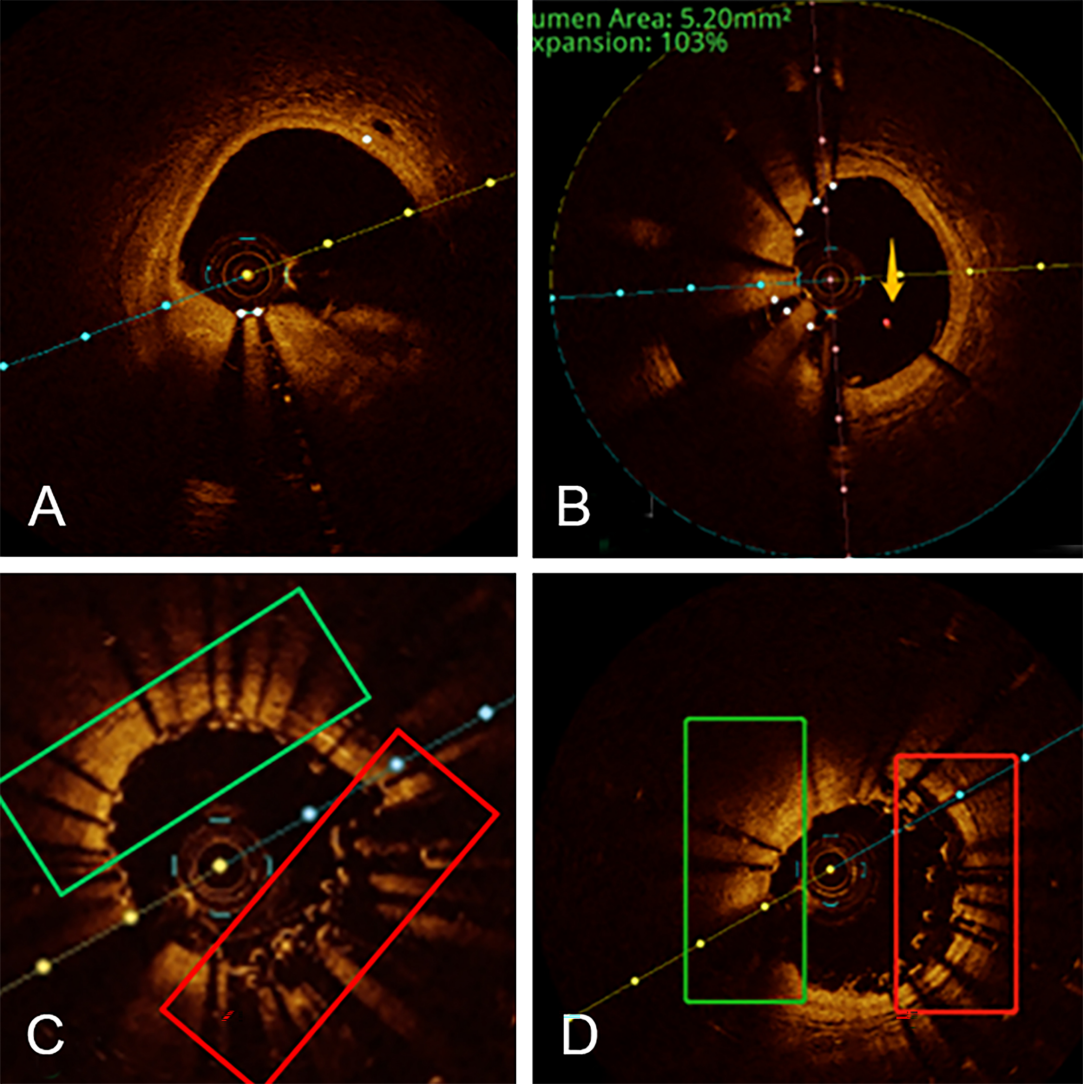
**Longitudinal stent deformation at the proximal segment of the left main coronary artery caused by a stuck optical coherence tomography (OCT) catheter**. (A) Before the catheter being stuck, OCT shows the possibility of dissection under the condition of the guidewire artifact. (B) Under the condition of no guidewire support, OCT shows no obvious dissection in the distal part of the stent in the middle segment of the LAD and the yellow arrows show the malapposition of stent beam. (C) After the stuck OCT catheter was removed, OCT shows incomplete stent apposition. (D) After another stent was inserted into the mid-distal segment of the LAD and post-dilatation, OCT shows single-layer stent beam in the 9 o’clock-2 o’clock direction and triple-layer stent mesh in the 3 o’clock-6 o’clock direction, indicating that is the area where stent was placed after passing through the previous stent mesh. LAD, left anterior descending artery; OCT, optical coherence tomography.

Elective transradial coronary angiography (CAG) showed borderline lesions and suspicious niche in the distal segment of the left main (LM) coronary artery, borderline lesions in the proximal to the middle segment of the left anterior descending artery (LAD), and diffused stenosis in the proximal to the middle segment of the circumflex artery (LCX) (up to 90%), but no significant stenosis was observed in the right coronary artery (Fig. 1A,B). OCT (75 mm pullback, St. Jude Medical, Little Canada, MN, USA) guided stents implantation (6F EBU3.5 guiding catheter, RUNTHROUGH in LAD and SION in LCX) was sequentially performed in the proximal LCX (3.0 × 29 mm, Promus PREMIER, Boston Scientific, Marlborough, MA, USA) and LM-LAD (2.75 × 33 mm, 4.0 × 18 mm, Promus PREMIER, Boston Scientific, USA). The proximal segment of LCX stent was about 2 mm away from the ostial of LCX indicating no connection between stents of LAD and LCX. Angiography showed complete stent apposition with TIMI grade III (Fig. 1C). OCT pullback of the LAD showed stent malapposition and stent dissection at the distal segment of the LAD. However, the presence of the guidewire artifact posed challenges in accurately measuring the depth, length, and angle of the dissection (Fig. 2A). Therefore, a re-pullback of OCT was performed to evaluate whether a bailout stent was required at the distal segment of the LAD stent during which the guidewire supporting the OCT catheter was retracted to the proximal LAD in order to minimize any potential artifact influence (Fig. 1E). Scanning revealed incomplete stent apposition at the distal segment of LAD stent and no bailout stent was needed (Fig. 2B).

In retracting the wireless OCT catheter, it stuck and could not be removed. Several attempts to advance, rotate, and withdraw the OCT catheter failed (Fig. 1F). After repeatedly adjusting the OCT catheter, it popped out of the artery together with the guiding catheter. The OCT catheter creased in the distal second marker segment.

Next, the guiding catheter was reintroduced. The angiography showed that the stent at the proximal segment of the left main coronary artery was shortened, and the “wedding ring sign” was observed (Fig. 1H). In addition, the lumen at the edge of the stent and inthe middle segment of the LAD decreased in size, with suspected dissection or thrombus (Fig. 1D). A bailout 2.5 × 15 mm stent (Promus PREMIER, Boston Scientific, USA) was implanted to overlap the distal part of the stent in the middle segment of the LAD, based on the finding of dissections, white thrombus and a decreased lumen were observed with a new OCT catheter (Fig. 1I). The OCT showed that the stent was well apposed to the wall after post-dilatation, and no dissection was observed in the distal segment of the LAD (Fig. 2C,D). OCT also revealed LSD at the proximal segment of the left main coronary artery, accompanied by stent edge dissection and stent malapposition. The 4.0 mm × 8 mm post-dilatation balloon (Quantum Maverick, Boston Scientific, USA) was reintroduced to post-dilate the stent at the left main coronary artery at 20 atmosphere (atm). The length from the ostium of the LAD to the stent of the LM shortened from 12.9 mm before LSD to 8.1 mm on OCT (Fig. 1G,H,J,K).

Tirofiban was given postoperatively, and no acute stent thrombosis occurred. In one-year follow-up, the patient did not experience any episode of angina attacks or acute myocardial infarction.

## 4. Literature Review

LSD refers to the distortion of the coronary stent along its longitudinal axis, either shortening or elongation. In brief, LSD occurs when the implanted coronary stent undergoes local deformation along its long axis, resulting in a variation exceeding 2 mm [3]. It is a rare complication of coronary stent interventions, with an incidence ranging from 0.2% to 0.68% [1, 2]. LSD can be visualized in CAG as a ring-like image caused by the overlapping stent beams and shortening along the long axis of the stent. This phenomenon has been described in literature as the “organ effect” or “accordion effect”. However, it is important to note that the incidence of LSD may be underestimated due to the limitations of CAG in diagnosing this condition. The ability of X-rays to penetrate different coronary stents can vary, and the clinical experience of physicians also plays a role in accurate diagnosis. In a study of coronary computed tomography (CT) angiography [22], the incidence of LSD was found to be 2.6%. Furthermore, the incidence of LSD may increase if IVUS or OCT is routinely performed after coronary stenting. If LSD is suspected after a detailed analysis of angiography after stenting, further diagnostic procedures such as IVUS or OCT should be considered to provide a more definitive diagnosis and guide subsequent interventional management [6, 7].

Compared to the first-generation drug-eluting stents (DES), the second-generation stents are thinner, resulting in reduced longitudinal strength and a 13-fold higher risk of LSD [22]. Among the second-generation DES, the Promus Element stands out with the least longitudinal compression strength due to its thin platinum-based stent and the offset peak-to-peak stent design, featuring two connecting rod racks [2, 23]. Studies conducted by Prabhu *et al*. [23] and Ormiston *et al*. [24] have demonstrated that the Promus Element has the highest incidence of LSD among second-generation drug-coated stents.

Approximately 89% of LSD cases can be attributed to the following mechanisms. Firstly, LSD may occur during the insertion or removal of interventional devices such as balloons, guidewires, rota devices, and IVUS catheters, as these devices can exert pressure on the stent. Secondly, it can occur during the removal of interventional devices when the guide catheter or extension catheter applies relative forces that squeeze the stent. A study by Rhee *et al*. [4] focused on predicting LSD and its clinical outcomes and identified several independent risk factors for LSD, including lesions in the LM or its opening, treatment of bifurcation lesions with double stents or jailed balloon, interventions performed at a distant part of the stent, IVUS examination, and postdilatation. Other related studies have also highlighted the potential contribution of interventional therapy for bridging vessels, jailed guidewires, pressure guidewires, rota wires, protection devices for the distal vascular segment, and non-slip element (NSE) balloons to the occurrence of LSD. However, there have been no reports in the literature of OCT catheters leading to LSD [3, 25]. Additionally, the use of NSE balloons increases the risk of LSD.

Chung *et al*. [22] concluded that LSD caused by DES was not associated with the development of major adverse cardiovascular events. However, it is important to note that this finding may be influenced by the limited number of patients included in the study. In contrast, other studies have reported that not all cases of LSD result in benign symptoms, with 14% of patients experiencing serious adverse events. These adverse events can include the need for heart bypass surgery due to the inability to remove the lodged medical device after LSD [8], acute stent thrombosis, subacute stent thrombosis, very late stent thrombosis [9], ISR, and death. Limited clinical studies have suggested that re-stenting or postdilatation management after the occurrence of LSD may reduce the incidence of adverse events associated with LSD. Based on some expert experiences, whether “rota ablation” of the deformed stent allows for better wall apposition of the additionally placed stent and further reduces adverse events in LSD remains unclear. Therefore, more reliable clinical study data is needed to address these aspects. Additionally, there is a lack of strong clinical research evidence regarding whether patients with LSD require longer dual antiplatelet therapy (DAPT) or lifelong DAPT. It is worth noting that very late in-stent thrombosis has been reported in patients with LSD even after one year of DAPT. In the absence of clinical evidence, endovascular imaging techniques such as OCT can be utilized to assess stent neointimal coverage, stent apposition, and dissection. This information can help guide the selection of appropriate antithrombotic drug therapy for individual patients (Table 1).

This rare case of a stuck OCT catheter, which causes LSD of the LM, was primarily due to the sharp protrusion of the non-guidewire-track-supported OCT catheter at both ends of the guidewire exit. The sharp protrusion was stuck in the tortuous middle segment of the anterior descending artery and stent beam with incomplete apposition, resulting in a stuck OCT catheter. Despite repeated attempts to forcefully remove the OCT catheter, the condition worsened, resulting in incomplete stent apposition in the middle segment of the anterior descending artery and a coronary artery dissection, ultimately leading to acute in-stent thrombosis. Additionally, the withdrawal of the OCT catheter caused deep insertion of the guide catheter, which affected the stent in the proximal segment of the left main coronary artery, causing LSD and exposing the previously covered dissection. When attempting to re-deliver the guidewire to the anterior descending artery, it was unable to pass through the stent mesh due to severe incomplete stent apposition in the middle segment. As a result, dilatation and stent implantation were performed again in the middle to distal segments. Fortunately, further examination with OCT revealed that the dissection in the left main coronary artery did not penetrate deep into the smooth muscle layer and had a small angle. Therefore, there was no need for an additional stent in the proximal segment. The original stent in the left main coronary artery was post-dilated again using a post-dilatation balloon, and the patient received postoperative intensive antiplatelet therapy. The patient was followed up for one year, and no complications were observed.

## 5. Conclusions

Due to the absence of support from the guidewire track, the OCT catheter became angled at the exit, resulting in sharp protrusions on both sides. These protrusions became trapped by the stent beam, resulting in malapposition of the stent, which caused the OCT catheter to become lodged. This further exacerbated the issue of stent malapposition. Furthermore, LSD was observed in the proximal segment of the left main coronary artery, exposing the previously covered stent dissection.

This case was introduced to warn people of these conditions. LSD may occur when OCT is performed without guidewire track support, particularly in tortuous lesions, LM opening lesions, long lesions, and lesions with incomplete stent apposition. For patients at risk of LSD, physicians should maintain the guidewire coaxial, avoid inserting the guidewire deep into the muscle layer and using excessive strength, and place the guide catheter at a proper distance from the proximal segment of the stent. When they push in or remove the OCT catheter, stent balloon, and postdilatation balloons, an imaging device must be used, or the protection guidewire or balloon must be removed. When LSD is suspected, OCT or IVUS must be performed as accurately as possible to carry out re-implantation of the stent or re-postdilatation.

## Data Availability

Data and materials are available on request.

## Abbreviations

AL, Amplaze guiding; CABG, coronary artery bypass grafting; DAPT, dual antiplatelet therapy; DES, drug-eluting stent; ISR, in-stent restenosis; IVUS, intravenous ultrasound; LAD, left anterior descending artery; LCX, circumflex artery; LM, left main artery; LSD, longitudinal stent deformation; OCT, optical coherence tomography; ST, stent thrombosis; VST, very late stent thrombosis.
